# Antibiotics—From There to Where?: How the antibiotic miracle is threatened by resistance and a broken market and what we can do about it

**DOI:** 10.20411/pai.v3i1.231

**Published:** 2018-02-22

**Authors:** David M. Shlaes, Patricia A. Bradford

**Affiliations:** 1 Anti-infectives Consulting (retired), Stonington, Connecticut; 2 Antimicrobial Development Specialists, LLC, Nyack, New York

**Keywords:** Antibiotics, antibiotic resistance, Pneumonia, Endocarditis, bacteria

## THE MIRACLE

To fully appreciate the importance of antibiotics to everyday life, we must step back to the edge of the pre-antibiotic era when these lifesaving drugs were first introduced into clinical use. In the pre-antibiotic era skin and soft tissue infections such as cellulitis and erysipelas (frequently confused) were often deadly. These serious infections of the subcutaneous fascia could involve the lymphatics and spread systemically. In the pre-antibiotic era, bacteremia was common and mortality reached as high as 15%. The introduction of sulfonamides into clinical use in the 1930s changed that and the resulting mortality was reduced to 2% [1].

**Figure 1.**
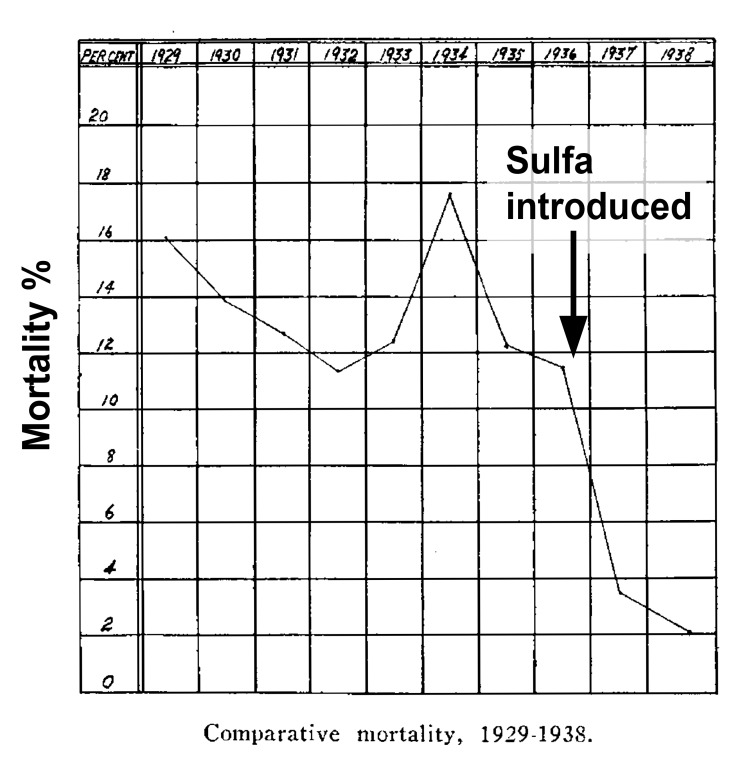
Mortality from Erysipelas Before and After the Introduction of Sulfonamide Antibiotic Use.

**Figure 2.**
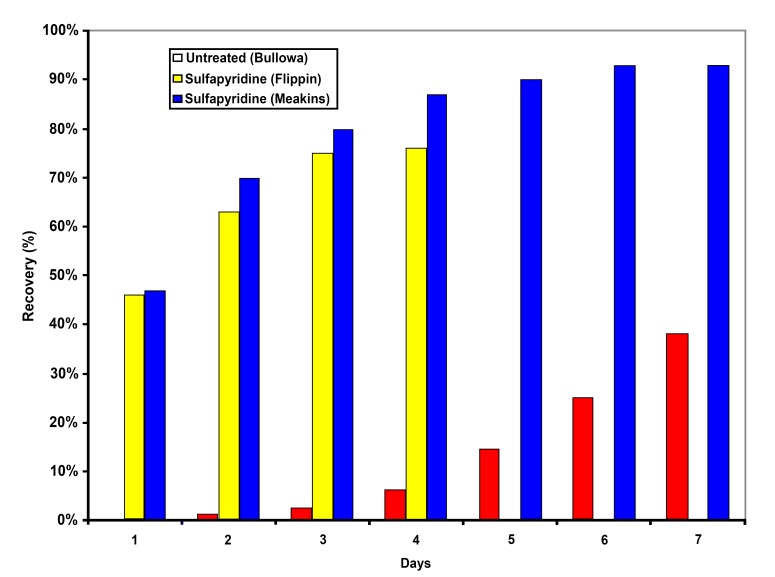
Antibacterial Treatment Effect on Clinical Recovery from Pneumococcal Pneumonia.

Pneumonia was also a deadly disease and when due to Streptococcus pneumoniae was referred to as the “captain of the men of death.” Approximately 30% of patients would succumb [2] to this infection. If the patient was bacteremic, 70-90% mortality was expected. Among survivors, only 30% were afebrile at one week. Therapy with penicillin reduced the mortality of bacteremic pneumonia to 17% [3]. Sulfonamide therapy resulted in 70-80% of patients being afebrile within three days [2], [4–6] (Figure 2). These are dramatic, some would say miraculous, treatment effects.

**Figure 3.**
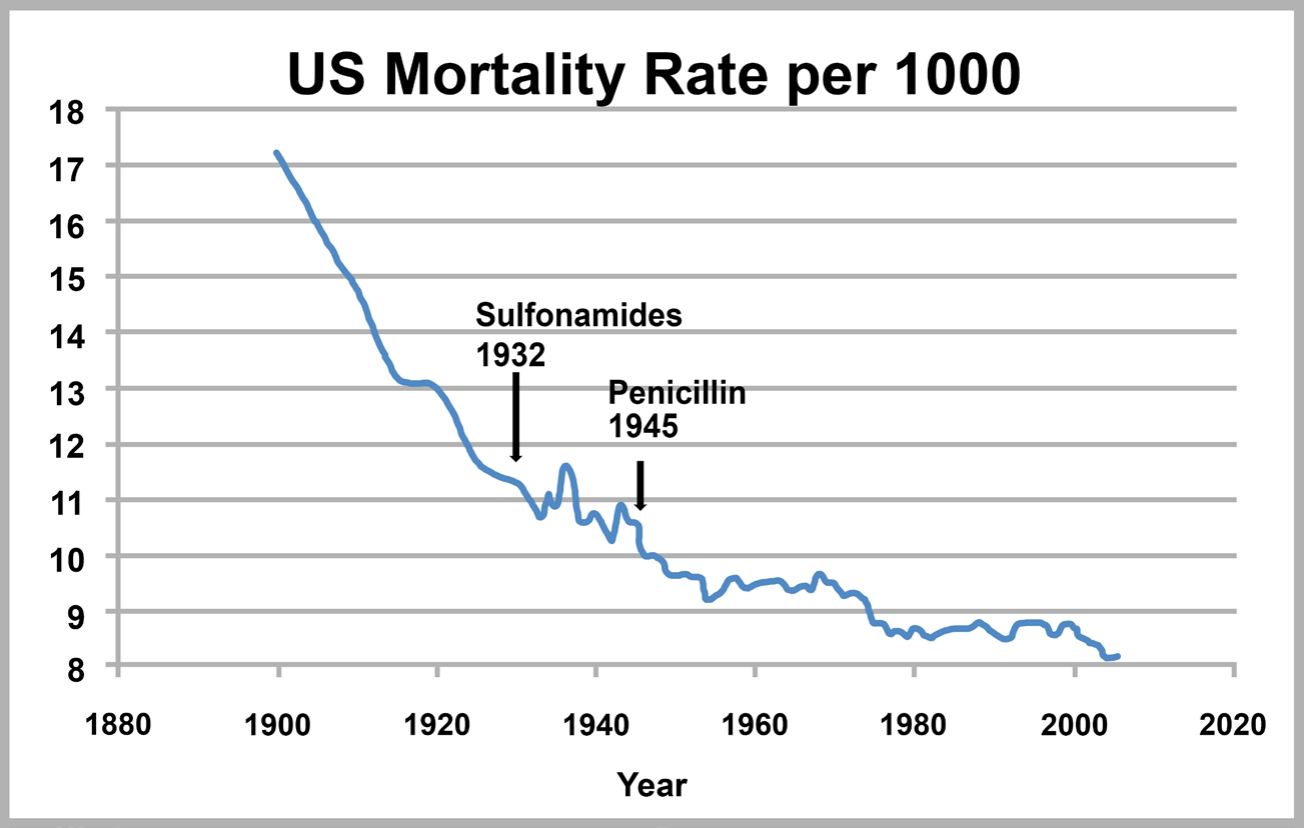
US Infectious Disease Mortality [10].

Endocarditis was a uniformly fatal infection before antibiotics [7]. Today, mortality rates overall are around 20% with antibiotics and with surgery when indicated [8]. Mortality associated with infectious diseases was declining by the beginning of the 20^th^ century, and this decline accelerated (with the exception of the 1918 influenza pandemic) with the introduction of sulfonamides and penicillin in the 1930s and 40s (Figure 3) [9]. Truly, antibiotics are a miraculous resource.

## THE THREAT

Today the miracle of antibiotic therapy is threatened by bacterial resistance that is emerging faster than we can discover, develop and market new drugs. This situation is untenable and is leading to severe consequences including increased morbidity and mortality and their associated societal costs. The evidence of increasing antibiotic resistance on a global scale is manifest.

Bacterial pathogens are developing resistance to all antibacterial agents in clinical use. Antimicrobial resistance (AMR) complicates the treatment for bacterial infections, resulting in concurrent use of multiple antibiotics, prolonged courses of therapy, and long hospitalizations. The Antimicrobial Review, published in 2016, estimated that currently 700,000 people/year worldwide die of infections caused by antibiotic-resistant pathogens and, by the year 2050, 10 million lives will be at risk annually if solutions to slow the emergence of drug resistance are not found [11]. In December 2017, a group of experts commissioned by the World Health Organization (WHO), assembled a list of 20 bacterial pathogens that are a major threat to public health because of the decreased efficacy of currently available antibacterial therapies due to resistance [12]. The criteria used to place organisms on this list included the resistance threat of the pathogen, available therapies, and new antibiotics in the development pipeline. These organisms were then stratified into three priority categories (critical, high, and medium) reflecting levels of concern and unmet needs [12].

**Table 1.** WHO priority list of antibiotic resistant bacteria

Priority Grouping	Organism or family	Resistance
1: Critical	*Acinetobacter baumannii*	carbapenems
	*Pseudomonas aeruginosa*	carbapenems
	Enterobacteriaceae*	carbapenems, third-generation cephalosporins
2: High	*Enterococcus faecium*	vancomycin
	*Staphylococcus aureus*	methicillin, vancomycin
	*Helicobacter pylori*	clarithromycin
	*Campylobacter* spp.	fluoroquinolone
	*Salmonella* spp.	fluoroquinolone
	*Neisseria gonorrhoeae*	Third-generation cephalosporins, fluoroquinolone
3: Medium	*Streptococcus pneumoniae*	penicillin
	*Haemophilus influenzae*	ampicillin
	*Shigella* spp.	fluoroquinolone

*Includes *Citrobacter* spp., *Enterobacter* spp., *Escherichia coli, Klebsiella* spp., *Proteus* spp., *Providencia* spp., *Morganella* spp., and *Serratia* spp.

Adapted from [12]

## PRIORITY: CRITICAL

### Carbapenem-resistant *Acinetobacter baumannii*

**Figure 4.**
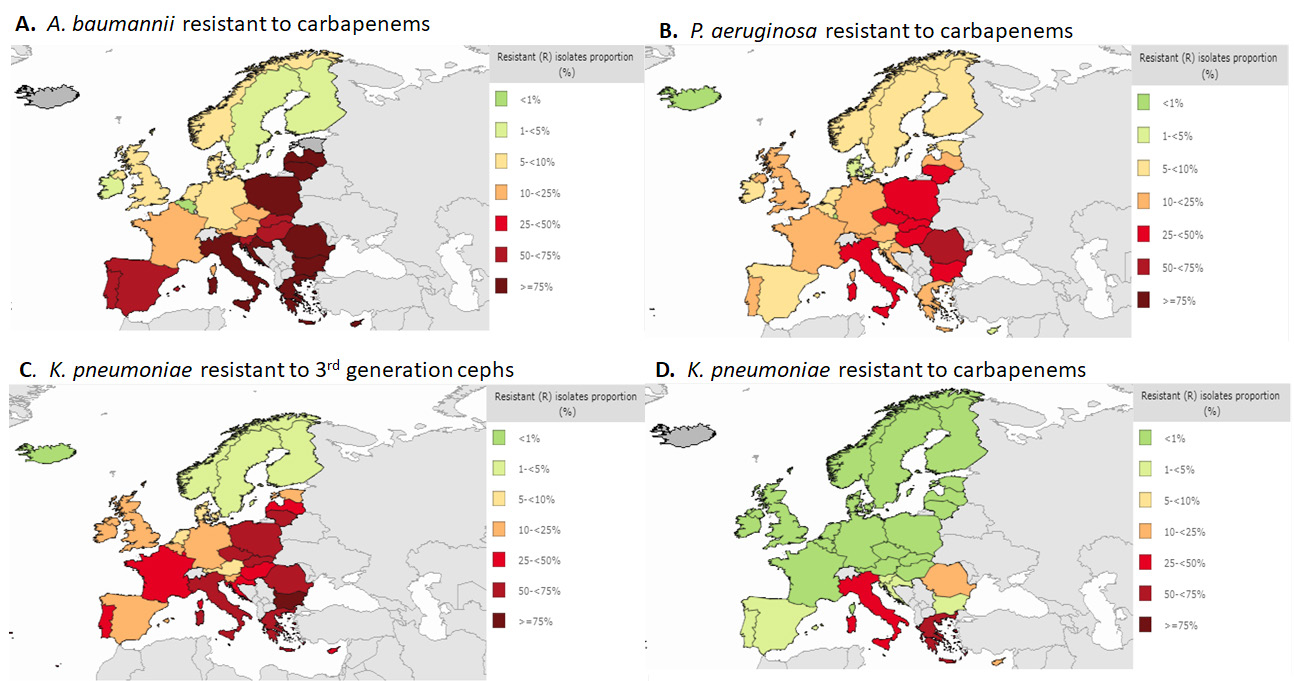
Percentage of antimicrobial resistance in Europe in 2015 [18].

Among Gram-negative pathogens, *Acinetobacter baumannii* has become increasingly prevalent in many intensive care units (ICUs) following the increased usage of carbapenems, especially among patients with mechanical ventilation [13]. Carbapenem-resistance in *Acinetobacter* spp. is caused by multiple mechanisms, involving several kinds of β-lactamases, most recently including the metallo-β-lactamases [14, 15]. In resistance data compiled in 2015 by the European Centre for Disease Control and Prevention (ECDC), more than 75% of *Acinetobacter* spp. are resistant to carbapenems in Mediterranean region countries (Figure 4A). The concern with carbapenem-resistant *Acinetobacter* spp. is compounded by the observation that many of these strains have also become resistant to colistin [16]. This results in extremely limited treatment options for patients infected with these organisms that include some creative combinations that involve ampicillin-sulbactam and tigecycline [17].

## CARBAPENEM-RESISTANT *PSEUDOMONAS AERUGINOSA*

*Pseudomonas aeruginosa* is one of the most commonly isolated pathogens that cause nosocomial infections, frequently among patients in intensive care units (ICU) [19]. *P. aeruginosa* has been associated with resistance to all known antibiotics, is often found to be multi-drug resistant (MDR) accounting for 30-50% of isolates [20, 21]. Infections with drug resistant *P. aeruginosa* lead to increased morbidity and mortality because the treatment of these infections is frequently empirical, thus antibiotic resistance in these isolates raises the likelihood of administering inappropriate initial therapy [22]. ECDC data showed that 5-24% of *P. aeruginosa* in most Western European countries were resistant to carbapenems, whereas in the Mediterranean countries and some Eastern European countries, resistance is 25-74% (Figure 4B). In recent years, the production of both serine- and metallo-based carbapenemases has become a major threat in infections caused by *P. aeruginosa,* as β-lactams are generally included in empiric combination therapies [23, 24]. Outbreaks of nosocomial infection due to this organism may be large and sustained, despite the adoption of infection control measures [25].

## EXPANDED-SPECTRUM CEPHALOSPORIN AND CARBAPENEM-RESISTANT ENTEROBACTERIACEAE

Resistance to expanded-spectrum cephalosporins and carbapenems among Enterobacteriaceae is primarily mediated through the presence of one or more β-lactamases and represents a growing public health crisis that threatens to make many serious infections among hospitalized patients “untreatable” with currently available antibiotics [26]. The widespread use of broad-spectrum cephalosporins has accelerated the emergence of extended-spectrum β-lactamase (ESBL)-producing *Escherichia coli* and *Klebsiella pneumoniae* that are now endemic in many hospitals worldwide [26].

Resistance data from ECDC showed 50-75% resistance to expanded-spectrum cephalosporins among isolates of *K. pneumoniae* in many European countries (Figure 4C). Although hospitalization is a major risk factor for infections caused by ESBL-producing Enterobacteriaceae, long-term care facilities, mostly comprised of elderly patients, have become recognized as an important reservoir of infection [26]. A recent study showed that 14.7% of nursing home residents in Germany were colonized with ESBL-producing *E. coli* [28].

As a result of the widespread prevalence of resistance to expanded-spectrum cephalosporins, the reduced efficacy of these cephalosporins due to resistance has led to increasing use of carbapenem antibiotics for common health care-associated infections. This, in turn, has resulted in the predictable emergence of carbapenem-resistant Enterobacteriaceae [29]. The prevalence of carbapenem resistance among *K. pneumoniae* remains low in most European countries, yet it is present in 50-75% of isolates in Italy and Greece (Figure 4D).

Carbapenem-resistance in Enterobacteriaceae is mainly due to acquired β-lactamases with the ability to hydrolyze carbapenems and can be serine- or metallo-based. Among the serine-carbapenemases Klebsiella pneumoniae carbapenemase (KPC) has the largest distribution, first endemic in New York City, but now found worldwide [24, 30]. A widely publicized outbreak of KPC-producing *K. pneumoniae* was documented in a clinical trial unit at the National Institutes of Health in which 11 of 18 patients died as a result of their infections [31].

Even more worrisome are Enterobacteriaceae that are carbapenem-resistant due to the presence of a metallo-β-lactamase (MBL), which are found in multiple species of Enterobacteriaceae and can be found globally [23]. One such MBL, the New Delhi metallo-β-lactamase (NDM), originated in and is endemic to all parts of India and Pakistan, and has spread across the globe [23, 32]. Carbapenem resistance in Enterobacteriaceae significantly impairs clinical management of patients with serious infections, as the organism is often multi- or pan-resistant to many of the currently available first-line therapeutic options [26].

## PRIORITY: HIGH

### Vancomycin-resistant *Enterococcus faecium*

Vancomycin-resistant *Enterococcus* (VRE) *faecium* was first encountered in clinical isolates in Europe in 1986, followed the next year by isolation of VRE in the United States [33, 34]. In the US, VRE is predominantly found in hospitals, because of the high usage of vancomycin to treat MRSA [35]. Among Western countries, the prevalence of VRE is highest in North America, with VRE comprising 35.5% of enterococcal hospital-associated infections, coinciding with the high prevalence of MRSA in the USA [36]. In Europe, VRE is less prevalent than in the US, but appears to be increasing in frequency. The European Antimicrobial Resistance Surveillance System reported a VRE *faecium* prevalence of 8.3% in 2015 [37]. *E. faecium* is intrinsically more resistant to antibiotics, with more than half of nosocomial isolates in the US expressing resistance to ampicillin, vancomycin, and aminoglycosides [38].

### Methicillin and vancomycin-resistant Staphylococcus aureus

*S. aureus* strains became methicillin-resistant by the acquisition of the gene *mecA,* which confers resistance to all β-lactam antibiotics except for the anti-MRSA cephalosporins ceftaroline and ceftobiprole that can bind to PBP 2a [39]. In adults, the prevalence of MRSA carriage is 25-50% for the general public, although a higher level of colonization is observed in certain populations such as injection drug users or patients with long-term indwelling intravascular catheters. It is thought that healthcare workers may be an important reservoir for MRSA [40].

At present, many industrialized countries report that MRSA comprises at least 25-50% of *S. aureus* isolated in hospitals [41]. In Japan, high antibiotic use has led to increased MRSA burden over time [42]. At first, when cases of MRSA infection were identified in the community setting, investigation usually exposed a history of recent hospitalization; close contact with a person who had been hospitalized; or previous antimicrobial-drug therapy. Now, MRSA is becoming more common in the community setting as well [43]. Community associated (CA)-MRSA is now an established cause of skin infections in the community, often in outbreaks among players of competitive sports, especially athletes who play contact sports [44]. Patients with recurrent MRSA infections make multiple healthcare visits before a wound culture is obtained. Recurrence of infections might be avoided if physicians obtain cultures more routinely when athletes have infected wounds [45].

## EXPANDED-SPECTRUM CEPHALOSPORIN AND FLUOROQUINOLONE RESISTANT N. GONORRHOEAE

In 2008, WHO estimated that there were more than 100 million new cases of gonorrhea among adults worldwide, representing a 21% increase from their 2005 estimate. In resource-limited countries, this disease often goes undiagnosed and untreated due to suboptimal diagnostics and patient management, resulting in substantial unrecognized morbidity and hidden healthcare costs [46]. Following the acquisition of β-lactamase, which reduced the efficacy of penicillin, fluoroquinolones—in particular ciprofloxacin—then became the treatment of choice for gonorrhea treatment.

Clinical failures of fluoroquinolones were reported by 1990. Resistance and dissemination were initially reported in the Asia-Pacific Region, but then rapidly spread globally [47]. In the United States, fluoroquinolone-resistant strains were initially imported from Asia in 2000, became prevalent in Hawaii, and then subsequently spread first to the West Coast and then to the rest of the US [48]. By 2007, the fluoroquinolones were removed from the recommended treatment regimens for gonorrhea due to high levels of fluoroquinolone resistance [46].

Following the demise of fluoroquinolones, the expanded-spectrum cephalosporins became the drugs of choice to treat gonorrhea, but the usefulness of this class has also been undermined by resistance [49]. During the last two decades, gonococcal strains exhibiting resistance to expanded-spectrum cephalosporins initially emerged in Japan and were thought to have arisen as a result of suboptimal drug dosing [50]. Over 30% of gonococcal isolates in Japan are resistant to cefixime. This resistance has now spread worldwide, and has resulted in clinical failures [50–52].

Especially worrisome is the emergence of extremely drug-resistant (XDR) strains of *N. gonorrhoeae* that have high-level resistance to all expanded-spectrum cephalosporins combined with resistance to nearly all other available antimicrobials [53]. Many of these XDR strains were identified in high-risk, transmitting populations such as commercial sex workers or men having sex with men (MSM) [46]. Because ceftriaxone is the last option for “first-line” empirical therapy of gonorrhea, the emergence of XDR strains of *N. gonorrhoeae* may be signaling the beginning of an era of untreatable gonorrhea.

## PRIORITY: MEDIUM

### Penicillin non-susceptible S. pneumoniae (PNSP)

*S. pneumoniae* is a major cause of illness and death worldwide, causing acute otitis media (AOM), acute sinusitis, community acquired pneumonia (CAP), and meningitis [54]. Since the introduction of penicillin in the mid-1940s, the treatment of pneumococcal infections was primarily with penicillin and other narrow-spectrum β-lactam antibiotics. PNSP were found periodically during the late 1970s, but then rapidly emerged and disseminated soon after. During the years 1993 to 1994, the percentage of non-susceptible isolates was 14.1%, but increased to 25% by 1999 [52]. Antibiotic resistance in *S. pneumoniae* is now a global public health problem, although the incidence of PNSP differs by geographic region. In parts of Latin America and Asia, 60-89% of *S. pneumoniae* are resistant to penicillin [55].

The incidence and spread of PNSP worldwide took a dramatic downturn following the introduction of the pneumococcal 7-valent conjugate vaccine (PCV7) in the year 2000 that included the most common serotypes found in invasive pneumococcal disease. The incidence of PNSP in the common serotypes has declined since the introduction of the vaccine; however, subsequently the highly resistant serotype 19A became the predominant serotype among fully penicillin-resistant strains (PRSP). The prevalence of PNSP among pneumococcal isolates in pediatric patients varies by country: 91.3% in Taiwan; 85.8% in Korea; and 70.4% in Vietnam, compared to < 1-5% in the United Kingdom and Scandinavian countries [18, 56]. Daycare centers for infants and pre-school-aged children have been shown to harbor and permit the spread of resistant isolates likely related to the sharing of bodily secretions, and receipt of multiple courses of antibiotics in the first few years of life [57]. The problem of resistance among community acquired isolates of *S. pneumoniae* is compounded by the frequent resistance to macrolides, which are commonly prescribed for respiratory infections [58].

Antibiotic resistance is of great concern in every country in the world. As noted by the priority list from the WHO, there are a number of bacterial pathogens for which treatment options are very limited, or in some cases nonexistent. We are on the precipice of returning to the pre-antibiotic era.

## WHY ARE WE ON THE EDGE OF THE CLIFF?

The proximate cause of bacterial resistance to antibiotics is use in humans and in agriculture and animal husbandry. There have been numerous attempts to control inappropriate use; some efforts have been successful and some have resulted in declining rates of resistance in controlled studies [59, 60]. But we must recognize the limitations of controlled use. Firstly, stewardship campaigns have not always been successful. Inappropriate antibiotic use in the US, especially in the community, remains rampant [61]. Limiting the use of medically important antibiotics in agriculture and in animal husbandry has also had only limited success. Some countries such as Norway have virtually eliminated the use of medically important antibiotics as growth promoters and in the prophylactic treatment of animals to prevent infection [62]. China, on the other hand, has allowed rampant use of antibiotics, including last-line drugs such as colistin as growth promoters [63]. This has resulted in the emergence of plasmid-mediated resistance to colistin that has spread to human populations. Second, even if we are successful in our efforts to reduce inappropriate antibiotic use, the remaining appropriate use will still select for resistance, albeit, in principle, at a slower rate.

Resistance is becoming a crisis today because, unlike in previous decades, our antibiotic pipeline is weak [64]. In the past, there were always new antibiotics being introduced to the market that allowed us to stay ahead of emerging resistance. Examples of this include the anti-pseudomonal cephalosporins like ceftazidime, the carbapenems for treatment of infections caused by organisms expressing extended spectrum β-lactamases, and the oxazolidinones and daptomycin for vancomycin-resistant enterococcal infections. Today's pipeline includes only five drugs in late-stage development that target high-priority resistant pathogens [12, 65–67]. Given the risks of failure, this is insufficient.

Several factors have led to this current critical state. The discovery of new antibiotics, especially those that can overcome intrinsic resistance mechanisms present in Gram-negative bacilli is challenging. In the early days of antibiotic discovery, relatively simple screening of soil samples for bacteria or fungi producing antibacterial substances was a fruitful endeavor. Now, identifying new, tractable chemical leads has become enormously challenging [68]. At Glaxo-SmithKline (GSK), a total of 67 high-throughput screens were undertaken from 1995 to 2001 against various essential gene targets in bacteria [68]. Only 16 of these screens identified hits. Of these 16 screens, only five resulted in lead compounds. None of these lead compounds became marketed antibiotics. In addition, empiric screening such as that used in the 1950s was also carried out and yielded no lead compounds. This experience remains typical for the industry today.

For many years, the regulatory system for antibiotics in the US was essentially nonexistent [9]. This changed in 2012 when the FDA revised their approach to antibiotic development in the face of a virtually empty pipeline—especially for antibiotics for the treatment of pneumonia [69]. The GAIN Act in the US helped in this regard, charging the FDA with providing feasible pathways for antibiotic development. The evolution of the US regulatory environment for antibiotics has been extensively reviewed elsewhere [70–72]. The regulatory pathways available for antibiotics today are generally feasible and often very streamlined when compared to those of previous years. Most no longer view the regulatory situation in the US or Europe as a disincentive for antibiotic development.

The general financial and competitive environment for the pharmaceutical industry has also been problematic. There have been two linked issues. First, the overall expense of bringing new drugs to market and the number of companies competing for market share have led to a pace of consolidation never before seen in other industries. A number of us carried out a study near the turn of the last century examining existing large pharmaceutical companies and the smaller companies that had been subsumed by these companies either through merger or acquisition [73]. For the six companies where we could identify clear records going back 20 years, we were able to document that they were derived from 70 smaller companies over those years [9]. This represents a 90% consolidation. When we then consider that two of those companies have been acquired since we carried out our study, this reflects a 95% consolidation in the last 30 years.

This consolidation is relevant to antibiotic research. When companies acquire or merge, they eliminate duplication seeking “synergy.” Synergy is code for staff reductions and other cost-cutting measures. If two companies with antibiotic research groups merge, it is likely that only one will survive. This consolidation has eroded the breadth of scientific pursuit of new antibiotics.

There is a broken market for antibiotics. Antibiotics are the black sheep of the pharmaceutical industry. Global sales of biopharmaceuticals exceed $900 billion per year [73]. The global antibiotics market is about $40 billion. Some of these sales represent generic drugs. Branded antibiotic sales account for about $28 billion of the total pharmaceutical market or about 3%. An examination of sales of recently approved antibiotics is even more dramatic (Table 2). For antibiotics marketed since 2009, total annual sales range from $9-119 million. These sales are dwarfed by the several billions of dollars in sales garnered by new drugs in the areas of diabetes and oncology, for example.

**Table 2.** Sales of recently marketed Antibiotics [74].

Antibiotic	Year of US Regulatory Approval	2015 Sales (millions) USD)
Ceftazidime-avibactam	2015	35.8
Tedizolid	2014	37
Dalbavancin	2014	20.3
Oritavancin	2014	9.1
Fidamoxicin	2011	39.8
Ceftaroline	2010	118.5
Telavancin	2009	9.4

These factors led to an abandonment of antibiotic research by large pharmaceutical companies that began at the turn of the last century. Whereas all large pharmaceutical companies were engaged in antibiotic research in 1990, only six large companies remain engaged today [73]. This abandonment adds significantly to the loss of researchers in the area.

Several key factors account for the “broken antibiotics market.” Possibly because antibiotics were among the first drugs to be introduced by the pharmaceutical industry, the approval of generic forms of older drugs occurred early. This led to a disconnect between the real value of antibiotics and their market value. According to a recent report by the National Academies of Science, Engineering and Medicine, [73], “*the median monthly cost of cancer drugs at the time of FDA approval increased from approximately $1,500 in 1965 to $150,000 in 2016, stated in constant 2014 dollars … The complexity of these issues is noted in one study that found that the average price of an episode of treatment using anti-cancer drugs is $65,900 and results in an average survival benefit of 0.46 years (not quality-adjusted).”* At the same time, the market for life-saving antibiotics that are far less costly (see below) has withered. Although this contrast may be greater in the US than in other countries, the principle is a global one.

Another important factor is simply one of patient numbers. A market analysis by Barrett illustrates this point [75]. This analysis was carried out in 2012 and focused on antibiotics targeting very specific bacterial infections that might be candidates for a small, focused development program. At that time, based on data contained in a report from Decision Resources and from the T.E.S.T. surveillance database, he could identify 295,000 individual infections caused by *A. baumannii*. He then estimated that about one-third of these (or 103,000 instances) were due to strains resistant to at least three classes of antibiotics. If we assume that a new antibiotic specific for *Acinetobacter* spp. would be used to treat a very generous 50% of these patients, to garner $500 million in revenues the price for a new antibiotic would have to be almost $5,000 per course of therapy. When Barrett carried out the same analysis for multiply resistant *P. aeruginosa* infections, he could only identify 54,000 patients. This would then lead to a price of $20,000 per course of therapy for a Pseudomonas-specific antibiotic. At the Pew Charitable Trusts Meetings [76] where this was discussed, even higher prices were suggested based on a lower projected market share.

The most expensive antibiotic ever to be marketed prior to 2015 was linezolid at $1,800 per course of therapy. Linezolid's commercial success was probably based on two main reasons. During the pandemic of MRSA infections that started around 1982, between 30-60% of hospital isolates of *S. aureus* were methicillin-resistant. Linezolid was the first orally available antibiotic proven to be useful in the treatment of MRSA infections. Linezolid was also active against VRE infections and was approved at a time when there were no other therapeutic options for many patients infected with VRE. The case of linezolid can be compared with that of ceftazidime-avibactam, an antibiotic active against a broad spectrum of Gram-negative pathogens including key carbapenem-resistant strains. But such resistant strains are much less frequent than MRSA. Ceftazidime-avibactam was approved for use in the US based on a very small phase II dataset. As such, its label was originally restricted to use for those patients with few or no alternative therapies available. (Since its first approval, a much larger dataset was used to expand the ceftazidime-avibactam label). The wholesale price was set at $12,000 per course of therapy but most hospitals could purchase it for $8,000.

The North American sales so far for ceftazidime-avibactam have not been more than about $35 million per year, representing about 4500 patients treated in North America.

There is clearly a perversion of pricing in the antibiotics market. In this case, a very cheap, old, toxic and ineffective antibiotic like colistin is used in preference to a very expensive, non-toxic and more effective alternative based in large part on the price difference. Hospital pharmacies are reluctant to stock such expensive new antibiotics when there is a cheap generic alternative available [77]. Thus, even with its high price, it seems unlikely that, short of a widespread epidemic of resistant infections, ceftazidime-avibactam will ever recoup its costs of development much less make a profit for its owners.

Another factor contributing to the broken antibiotic market is antimicrobial stewardship. The principle of antimicrobial stewardship is to limit the emergence of resistance by assuring the appropriate use of antibiotics and limiting inappropriate use. From all points of view stewardship is a public health need and also provides for more efficient use of hospital resources [78, 79]. In fact, as noted above in the example of ceftazidime-avibactam, high prices for antibiotics help assure limited use. One way we should *not* approach fixing the market is by encouraging inappropriate use of antibiotics. One way we *should* approach the problem is to assure appropriate use of new agents even when their price is high.

## “FIXING THE BROKEN ANTIBIOTIC MARKET”

Given that we need a robust pipeline of new antibiotics to keep up with ever-emerging resistance, and given that there is no longer an attractive commercial marketplace for new antibiotics, we need to include market considerations in our overall approach to the problem of resistance. In fact, when one examines all the factors that have led to our current lack of a robust pipeline, the one area where we have made no progress is the broken antibiotic market. We have made enormous progress on the regulatory front with streamlined and feasible approaches for development available for antibacterials [68–70]. Progress has even been made in learning how to synthesize compounds that can penetrate the Gram-negative outer and inner membranes and avoid the multiple efflux pumps employed by these organisms [80]. But with a market that continues to struggle, companies continue to abandon the arena, as illustrated by the recent departure of The Medicines Company from the antibiotics business [81].

The broken antibiotics market has been the focus of a great deal of thought, resulting in an ever-increasing body of published work since the white paper from the Infectious Diseases Society of America (IDSA) in 2004 entitled Bad Bugs, No Drugs [82]. Recent analyses focused on the economics of antibiotics and antibiotic resistance in order to develop solutions. The Antimicrobial Resistance Review led by Goldman-Sachs economist James O'Neill provided a projection of the mortality and societal cost of resistant infections in 2050 assuming current trends continue unabated [11]. In that scenario, infections caused by resistant organisms would ultimately be responsible for 10 million deaths and a loss of $100 trillion in world Gross Domestic Product. This provides a framework for investing in the antibiotics market today to prevent this projected outcome.

Mossialos *et al.*, defined “push and pull” incentives for the antibiotic market in 2009 [83]. “Push” incentives would be those that would reduce the cost of research and development of new drugs such as provided by research grants and monetary and in-kind support for preclinical and clinical development. This is another area where we have made important progress. The Biomedical Advanced Research and Development Authority (BARDA) of the Department of Health and Human Services in the US has been providing significant levels of support for antibiotic projects in late stage clinical development for a number of years [84]. The Innovative Medicines Initiative in Europe fills a similar function. More recently, a collaboration between BARDA, The Wellcome Trust, the National Institutes of Health, the AMR Centre and others, provides support for promising preclinical research programs (CARB-X) [85].

Nevertheless, most authorities immediately recognized that this could never be enough because the costs associated with any single drug program are only a small portion of the overall costs of pharmaceutical research. Whereas a single program might cost $250 million dollars, the overall cost of delivering a new drug to market today is more like $2.6 billion [86]. The reason for this is the very high failure rate of pharmaceutical research. We estimate that fewer than 5% of projects initiated in pharmaceutical research labs ever result in a marketed product. Of those projects that make it to clinical trials in humans, less than 20% will make it all the way to the market [86]. These failures still cost money. The farther a program progresses, the greater the costs incurred. So, a true incentive for marketing a drug must take into account the costs associated with the failures as well as the successes. These considerations lead to the concept of “pull” incentives. “Pull” incentives provide for an infusion of money starting at the time of drug approval that assures the sponsor that there will be a return on investment in research and development.

Several models for such pull incentives have been proposed and analyzed. These involve a contractual relationship with the sponsor as opposed to a single lump sum payment upon drug approval [87–90]. This can assure that the sponsor continues to be responsible for drug manufacture, distribution, regulatory support, surveillance for resistance, and other key activities. These models include market entry rewards, an insurance model, and transferable patent exclusivity vouchers. All of these models seek to “de-link” sales volume from revenues to varying degrees by providing a return on investment upon drug approval. All also restrict the availability of such incentives to drugs targeting priority pathogens as set forth by the CDC in the US or the WHO. Most authorities agree that such an award should be from $800 million to $2 billion depending on the degree of de-linking desired and other factors [87–90].

In the insurance model, national or regional authorities would agree to purchase a quantity of the new drug at a negotiated price, guaranteeing a market [86]. This also guarantees that the government will have a supply of the new drug in case of an epidemic of resistant infection. Additional drug could also be purchased as needed. This incentive is easier to explain to people, as it is familiar to them because it resembles the way we handle firefighting personnel and equipment. We pay for them hoping we won't need them. This mechanism is already in use for drugs and vaccines for biothreat agents. BARDA recently purchased doses of two Ebola vaccines for the national stockpile, for example [91].

**Figure 5.**
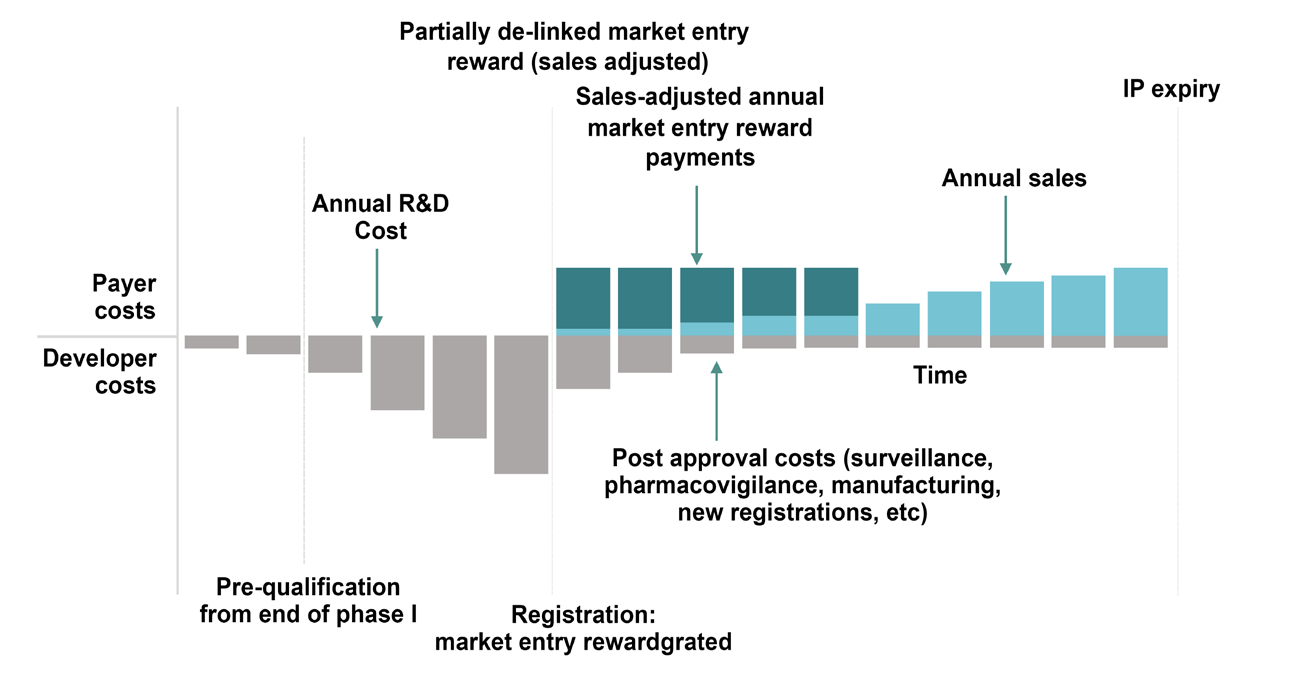
A partially de-linked market entry reward model [92].

Market entry rewards provide an upfront payment, extended over several years in most models, upon approval of an appropriate new antibiotic. In a fully delinked model, the payment usually provides for no sales or only minimal sales of the new product. In this case the payment would be higher than that for other models. On the other hand, even though sales volume and revenues are delinked, hospitals and others would still have to purchase the new antibiotic at a certain price. This approach helps assure appropriate antibiotic stewardship. In a partially delinked model such as that preferred by DRIVE-AB (Figure 5) [92], sales are allowed. This approach can provide for generic entries into the market at the end of exclusivity and therefore provides an advantage over fully delinked models.

Transferable exclusivity vouchers could be a powerful incentive [87]. In this approach, there is no government payment. Instead, the sponsor who succeeds in gaining approval for a new antibiotic active against priority pathogens would be allowed to extend the exclusivity of a drug of their choice from their portfolio of products. For example, AbbVie recently lost exclusivity for Humira, their $14 billion dollar per year product. For them, a few weeks or months of additional exclusivity for Humira would have resulted in hundreds of millions or billions of dollars in additional revenues. Because the voucher is transferable, if AbbVie had licensed an appropriate new antibiotic from a small biotech company, they would still receive the voucher. The biotech company would be rewarded by the purchase of their product by a large company like AbbVie. The major disadvantage of the voucher is that it will target those patients needing high-priced specialty drugs that tend to be the biggest sellers by delaying generic entries into the market. Some see the fact that this would not be a government payment as an advantage.

## CONCLUSIONS

Antibiotics are “miracle drugs.” They can cure disease in just a few days and, more importantly, save lives. Resistance to these drugs by pathogenic bacteria now threatens this miracle. Resistance is found in community-based pathogens such as *S. pneumoniae* and *N. gonorrhoeae* and in nosocomial Gram-negative pathogens such as *P. aeruginosa* and *A. baumannii*. This resistance forces us to use more and more of our “last line” antibiotics which, in turn, selects for resistance to those agents. At the same time, the broken antibiotic marketplace has left us in a situation where our pipeline of new antibiotics is precarious at best. To address this threat, we need to preserve the activity of the antibiotics we have today by controlling their use both for human disease and in the agricultural sector. Our most pressing need, though, is to fix the broken antibiotics market. Without addressing this critical problem, we can expect companies to continue to abandon antibiotic research. This, combined with continued consolidation within the industry, will further deplete our resource of antibiotic discovery researchers. This, in turn, will lead to a further deterioration in our already meager pipeline of new and desperately needed antibiotics. To fix the market, we need to assure a return on investment for those companies pursuing antibiotic discovery and development. “Push” incentives, those that reduce costs of research, have already been well established in the United States and Europe and are helpful. But these will not be sufficient because they do not take into account the capital costs of research failures. To address this problem and ultimately to assure a return on investment, “pull” incentives will be required. A number of models have been proposed for such incentives and, in our opinion, could perform as predicted. None have yet been adopted by any national or regional authority.
